# Supplementary material for: MetaRibo-Seq measures translation in microbiomes
Source: Nat Commun. 2020 Jun 29;11:3268. doi: 10.1038/s41467-020-17081-z (PMC7324362; doi:10.1038/s41467-020-17081-z)
Supplement: Supplementary file 10 — Supplementary Data 7 [file 41467_2020_17081_MOESM10_ESM.zip › File2/Confidence_VeryHigh_Taxonomy/163027_out.krona.html]

Javascript must be enabled to view this page.

members
magnitude
magnitudeUnassigned
count
unassigned
taxon
rank

163027\_out

8

superkingdom

SRS011134\_contig\_number\_51141
2
1
8

3
phylum
1224

class
1236
2

order
91347
1

543
family
1

1
genus
547

1
species
399742

SRS077127\_contig\_number\_18051

72274
order
1

135621
family
1

286
genus
1

1
237609
species

SRS015694\_contig\_number\_contig-100\_39239.39240

class
28211
1

356
order
1

family
41294
1

genus
374
1


SRS149075\_contig\_number\_contig-100\_7967.7968
1619232
species
1

1239
phylum
4

186801
class
4

186802
order
4


SRS056273\_contig\_number\_22660SRS075078\_contig\_number\_2208
1897045
species
2

family
541000
2

genus
946234
1

1
species
292800

SRS019693\_contig\_number\_contig-100\_148.68749

1

SRS098644\_contig\_number\_49870
2292270
species
